# Influence of low free thyroxine on progression of chronic kidney disease

**DOI:** 10.1186/s12882-019-1677-3

**Published:** 2020-01-30

**Authors:** Alexandre Barbosa Câmara de Souza, Marcia Fernanda Arantes, Roberto Zatz, Rosilene Motta Elias, Roberto Iglesias Lopes, Etienne Macedo

**Affiliations:** 1 Endocrinology Service, Hospital das Clinicas HCFMUSP, Universidade de São Paulo, São Paulo, Brazil; 2 Nephrology Service, Hospital das Clinicas HCFMUSP, Universidade de São Paulo, São Paulo, Brazil; 3 Universidade Nove de Julho, UNINOVE, São Paulo, Brazil; 4 Urology Service Hospital das Clinicas HCFMUSP, Universidade de São Paulo, São Paulo, Brazil; 5 Nephrology Division, Department of Medicine, University of California San Diego, San Diego, California USA

## Abstract

**Background:**

Hypothyroidism is highly prevalent in patients with chronic kidney disease (CKD) and has been associated with poorer clinical outcomes, including faster decline of kidney function. However, there is no consensus whether low free thyroxin (LFT) affects the rate of estimated glomerular filtration rate (eGFR) decline and how the presence of proteinuria influences the progression of renal dysfunction in hypothyroidism.

**Methods:**

We assessed thyroid status, proteinuria, and progression of eGFR by Modification of Diet in Renal Disease equation and CKD-EPI equation in a cohort of CKD patients followed in general nephrology clinics. We estimated the association of LFT levels, and the degree of proteinuria on progression of eGFR. We adjusted for other covariables: age, gender, body mass index, diabetes, hypertension, HbA1c, uric acid, cholesterol, and triglycerides levels..

**Results:**

One thousand six hundred ten patients (64 ± 15 years, 46.8% men, 25.3% diabetic) were included. At beggnining of follow up eGFR was between 45 and 60, 30–45 and 15-30 ml/min/1.73m^2^ in 479 (29.8%), 551(34.2%), and 580(36.0%) patients, respectively. LFT levels were available at initial evaluation in 288(17.9%) patients and 735(48.5%) had assessment of proteinuria (19.6% with LFT vs. 15.4% without LFT, *p* = 0.032). Median follow-up time was of 21 months, and 1223(76%) had at least 1 year of follow up. Overall, eGFR decline per month was − 0.05(− 0.26, 0.23) ml/min/1.73m^2^, reaching 1.7(1.3, 2.4) ml/min/1.73m^2^ by the end of study period. Similar results were obtained using CKD-EPI. Multivariable mixed linear analysis showed that proteinuria and age were independently associated with eGFR decline, with no effect of LFT, and no interaction between proteinuria and LFT. In patients without proteinuria, there was an improvement of eGFR despite the presence of LFT.

**Conclusions:**

We confirmed a faster rate of eGFR declined in patients with proteinuria. However, despite the pathophysiological rational that hypothyroidism can lead to increased rate of CKD progression, we failed to demonstrate an association between LFT and rate of CKD progression. We conclude that the benefit of hypothyroidism treatment in CKD patients needs to be evaluate in prospective studies.

## Background

During the embryonic period and after the maturation of the kidney, the thyroid gland influences the kidney’s growth and development. Thyroid hormones play an essential role in renal physiologic homeostasis through direct influence on the expression and activity of a number of ion channels and transporters [1].

Hypothyroidism is prevalent in patients with chronic kidney disease (CKD) and approximately 20% of patients with an estimated glomerular filtration rate (eGFR) < 60 ml/min per 1.73m^2^ will receive this diagnosis [2]. Hypothyroidism diminishes protein synthesis and cellular development, which leads to decreases in cell number, density, and size of the kidney [3]. It has been demonstrated that subclinical hypothyroidism might be a risk factor for faster decline of renal function [4–6], with possible effect on recovery or preservation of renal function with treatment [7, 8]. However, this finding are controversial and the effect of low free thyroxine (LFT) levels on eGFR decline in CKD patients has not been thoroughly investigated. Chaker L et al. [9] observed that hypothyroidism was associated with a lower risk of CKD in a prospective study with 5103 patients aged > 45 years with normal renal function. In a prospective cohort, in patient with normal kidney function, Zhan Y et al. found an increased incidence of CKD in patients with hypothyroidism after a median follow-up of 3.5 years [10]. In contrast, Mewese CL et al. [11], examining 72,856 individuals with overt hypothyroidism, subclinical hypothyroidism, and euthyroidism, concluded that low thyroid function was not associated with faster decline of renal function.

In patients with proteinuria, the hallmark of the accelerated progression of renal dysfunction, hypothyroidism contributes to transcapillary protein extravasation [12]. Thus, the presence of proteinuria may be a common mechanism or contributory factor for disease progression in CKD, what may explain, at least in part, the discrepancy on the results across previous studies that did not control for the degree of proteinuria. The primary objective of this study was to evaluate the association between LFT and an eGFR decline in patients with CKD with and without proteinuria.

## Methods

**Fig. 1:**
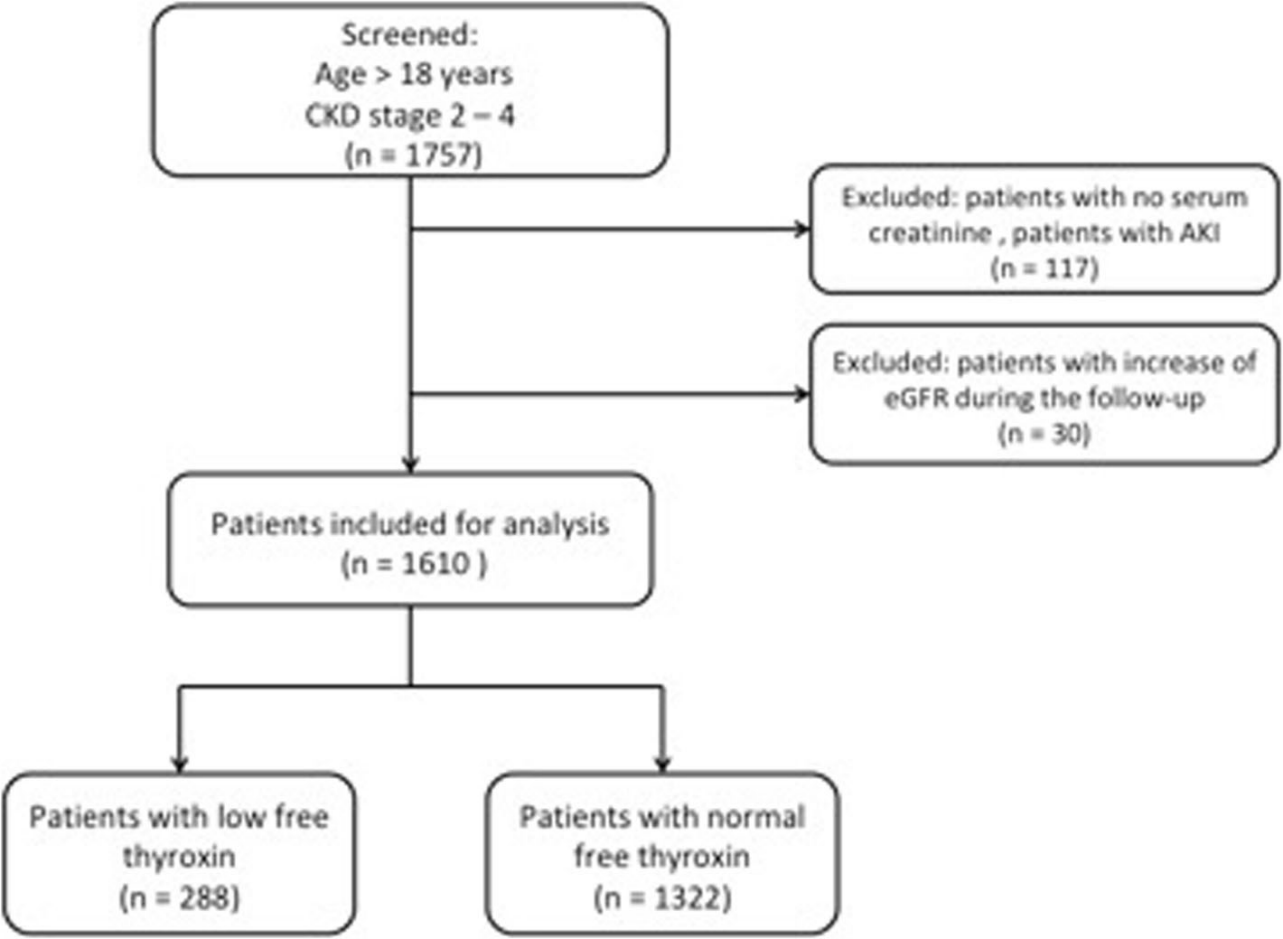
Flowchart of study. CKD, chronic kidney disease; AKI, acute kidney injury

A longitudinal analysis was performed using eletronic medical records from the Nephrology outpatient clinic of the Hospital das Clínicas, Universidade de São Paulo. We initially included 1757 adult patients followed in our clinic between September 2010 and September 2013, for whom serum creatinine (sCr), thyroid stimulating hormone (TSH) and free T4 were available. Other laboratory parameters such as proteinuria, lipid profile, uric acid, fasting glucose, and hemoglobin A1c levels were extracted. In all patients, kidney function was assessed at least twice and we exclude those with no sCr assessment within the first 6 months of initial assessment. To avoid including acute kidney injury (AKI) patients, we excluded patients with evidence of stage 2 AKI based on an increase in sCr of more than 200% between two consecutive measurements within 6 months [13]. Patients with an increase in eGFR greater than 1 ml/min/1.73m^2^/month during the follow-up period were also excluded. In the patients with hypothyroidism, the first assessment was at the time of diagnosis and at least once after 6 months. The final analysis included 1610 patients (Fig. 1).

Hypertension was defined according to the Eight Report of the Joint National Committee on Prevention, Detection, Evaluation, and Treatment of High Blood Pressure [14]. Diabetes was determined according to the American Diabetes Association guidelines [15]. Uric acid, triglycerides, total cholesterol, and fractions were assayed by enzymatic colorimetric method using automated equipment (BioSystems 200 Mindray® Model, Nanchan, China). LDL cholesterol was estimated by using the Friedewald equation [16]. Hyperlipidemia was defined as fasting LDL cholesterol concentration above normal limit or use of hiperlipidemic medication. Hyperuricemia was defined as values above the normal limit for men and women (7,0 and 6,0 mg/dl, respectively). Serum creatinine was assessed by the Jaffé technique. Hemoglobin A1c (HbA1c) was evaluated by high-performance liquid chromatography (HPLC). eGFR was calculated by the abbreviated Modification of Diet in Renal Disease (MDRD) equation [17] and CKD-EPI [18].

TSH (normal range 0.27–4.20 uIU/mL, detection limit 0.01 mU/l) and fT4 (normal range 0.93–1.70 ng/dL) levels were measured by a chemiluminescence immunoassay. We classified as LFT any measurement of fT4 lower than 0.93 ng/dL during baseline and at the follow-up (irrespective of whether hypothyroidism was primary or central) [19]. Patients were considered euthyroid if all free thyroxin and TSH levels were within the reference range during the follow-up period.

Proteinuria was defined by the presence the albumin/creatinine ratio (ACR) higher than 0.3 g/g [20].

The rate of renal functional decline was calculated using the formula: slope = (final eGFR) - (initial eGFR)/months of follow up, and was expressed as the median slope decline eGFR ([mL/min]/ [month/1.73m^2^]). We further classified patients according to eGFR decline/month above or below the cohort median decline as progressors and non-progressors.

### Statistical analysis

Data are presented as mean ± SD, median (25, 75) or as a percent, as appropriate. Normality distribution was verified by the Kolmogorov-Smirnov test. Comparison between patients with and without LFT as well as with and without proteinuria was done by unpaired t-test or Mann-Withney, for continuous variables and Chi-squared or Fisher for categorical variables, as appropriate. To compare longitudinal changes in eGFR across groups with and without proteinuria and with and without LFT, we performed a mixed linear model with repeated measures. The model was applied with a maximum likelihood estimation of variance components. The conditions evaluated were the presence of proteinuria, treated as a fixed factor, the presence of LFT also treated as a fixed factor and the interaction between these factors. Covariates in the model were age, gender, body mass index, and diabetes. The covariance was treated as AR (1) heterogeneous, which exhibited the lowest Akaikes’s Information Criterion (AIC).

In multivariable logistic regression analysis, we tested the factors associated with eGFR decline. Independent variables examined in the model were LFT, proteinuria, age, gender, BMI, presence of diabetes, and systolic blood pressure. *P* values < 0.05 were considered to be statistically significant. Statistical analyses were performed using SPSS version 21.0 (SPSS Inc., Chicago, IL, USA).

## Results

### Baseline

Clinical and biochemical characteristics of the patients are described in Table 1. The majority of patients were female; initial eGFR was between 45 and 60, 30–45 and 15-30 ml/min/1.73m^2^ in 479 (29.8%), 551 (34.2%), and 580 patients (36.0%), respectively. Hypertension was highly prevalent, whereas diabetes mellitus was found in 25.3% of patients. Although mean levels of total cholesterol were within normal range, 37% of patients presented hyperlipidemia, and 61% had hyperuricemia.

**Table 1:** Patient baseline clinical and biochemical characteristics at the study entry and according to quartiles of eGFR decline over time. (1st quartile represents the greater kidney disease progression and 4th quartile represents the less important kidney disease progression)

Characteristic	Entire group *N* = 1610	1st Quartile *N* = 405	2nd Quartile *N* = 511	3rd Quartile *N* = 294	4th Quartile *N* = 400	p
Age, years	64 ± 15	63 ± 16	65 ± 15*	66 ± 15*	64 ± 15	0.033
Male gender, %	46.8	48.5	55.2*	34.7	43.4	0.0001
Diabetes mellitus, %	25.3	30.4	23.9*	24.1*	22.7*	0.049
Hypertension, %	76.1	75.0	75.5	82.0	73.8	0.069
Systolic blood pressure, mmHg	136 ± 25	141 ± 29	135 ± 23*	136 ± 23	133 ± 25*	0.0001
BMI, kg/m^2^	27.9 ± 6.0	27.8 ± 6.5	28.2 ± 6.7	27.1 ± 4.8	28.1 ± 5.2	0.076
Proteinuria, %	48.5	64.6	45.1*	41.0*	42.3*	0.0001
eGFR, ml/min/1.73m^2^ (MDRD)	36 (26, 48)	36 (25, 48)	35 (24, 47)	34 (25, 46)	38 (28, 49)*	0.013
eGFR, ml/min/1.73m^2^ (CKD-EPI)	40.1 (28, 56)	23 (20, 23)	34 (31, 37)*	47 (44, 51)*	66 (60, 75)*	0.0001
Serum creatinine, mg/dl	1.88 ± 0.66	1.93 ± 0.71	1.89 ± 0.66	1.91 ± 0.69	1.79 ± 0.59*	0.015
eGFR decline, ml/min/1.73m^2^ (MDRD)	0 (−0.27, 0.24)	− 0.56 (− 0.91, − 0.36)	−0.07 (− 0.15, 0)*	0.12 (0.06, 0.17)*	0.53 (0.35, 0.91)*	0.0001
eGFR decline, ml/min/1.73m^2^ (CKD-EPI)	0 (−0.31, 0.26)	−0.67 (−1.15, − 0.67)	0.09 (− 0.19, 0)*	0.13 (0.06, 0.20)*	0.67 (− 0.40, 1.43)*	0.0001
TSH, uIU/ml	2.6 (1.6,4.1)	2.7 (1.7, 4.1)	2.6 (1.6, 4.3)	2.5 (1.6, 3.8)	2.6 (1.6, 4.3)	0.473
Free thyroxin, ng/dL	1.18 ± 0.28	1.18 ± 0.29	1.18 ± 0.27	1.20 ± 0.26	1.20 ± 0.31	0.751
LFT, %	17.9	20.0	17.4	13.9	19.2	0.176
Thyroxin replacement therapy, %	19.7	19.9	19.1	40.0	25.0	0.689
Hemoglobin A1C	6.4 ± 1.6	6.7 ± 1.8	6.3 ± 1.5*	6.3 ± 1.4*	6.3 ± 1.6*	0.007
Total cholesterol, mg/dl	191 ± 57	193 ± 60	189 ± 57	190 ± 44	194 ± 61	0.620
HDL cholesterol, mg/dl	50 ± 17	50 ± 16	50 ± 16	51 ± 16	50 ± 17	0.934
LDL cholesterol, mg/dl	111 ± 47	112 ± 52	109 ± 44	110 ± 36	112 ± 53	0.718
Triglyceride, mg/dl	158 ± 100	164 ± 106	154 ± 105	147 ± 77	164 ± 102	0.066
Uric acid, mg/dL	7.2 ± 2.0	7.5 ± 2.1	7.2 ± 1.9	7.0 ± 1.8*	7.2 ± 2.1	0.026
Hyperuricemia, %	61.3	64.1	57.9	61.9	62.5	0.271

Values are mean SD or median (25,75) unless indicated otherwise. * *p* < 0.05 vs. first quartile

*BMI* body mass index, *eGFR* estimated filtration rate, *CKD* chronic kidney disease, *TSH* thyroid stimulating hormone, *LFT* low free Thyroxin

Two hundred and eighty eight patients (17.9%) had LFT at baseline, and of these, 113 (39.2%) were receiving thyroid hormone replacement. Patients with LFT were younger (61.4 ± 15.3 vs. 65 ± 15.0 years, *p* = 0.0001) and presented high levels of total cholesterol (199 ± 62 vs. 190 ± 55 mg/dl, *p* = 0.013) and triglycerides (176 ± 100 vs. 154 ± 96 mg/dl, *p* = 0.013). There was no other clinical or biochemical difference between patients with and without LFT.

Albumin-creatinine ratio (ACR) was in 1514 patients, and it was above 0.3 g/g in 735 (48.5%). Proteinuria was more frequent among patients with LFT than without LFT (19.6% vs. 15.4%, *p* = 0.032).

**Fig. 2:**
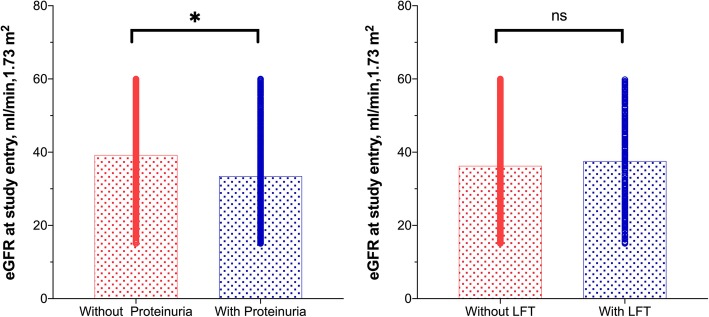
Cross-sectional association between estimated glomerular filtration rate (eGFR) at the study entry according to the presence of proteinuria and low free thyroxin (LFT) Estimated glomerular filtration rate (eGFR); LFT, low free thyroxin

Fig. 2 shows a box-plot graphic on a cross-sectional association between thyroid function and eGFR, and the effect of proteinuria. Patients with proteinuria presented lower eGFR than those without proteinuria (*p* < 0.0001). There was no difference in baseline eGFR in patients with and without LFT (*p* = 0.114).

### eGFR decline over time

The median follow up time was 21 months, and 1223 (76%) had at least 1 year of follow up. Overall, eGFR decline per month was − 0.05 (− 0.26, 0.23) ml/min/1.73 m^2^.

Results of multivariable mixed linear analysis adjusted for age, gender, diabetes, and BMI are shown in Table 2. Proteinuria and age were the only factors independently associated with an annual decline of eGFR. There was no interaction between proteinuria and LFT. Results remained unchanged after adjustment for thyroxin use. Furthermore, a sensitive analysis excluding patients on hormone replacement therapy yeld similar results, confirming that proteinuria but not LFT was associated with eGFR decline.

**Table 2:** Effects of two independent factors “proteinuria” and “low free thyroxin (LFT)” in the loss of renal function, evaluated by decline of estimated glomerular filtration rate (eGFR)

Variable	*p* value
Proteinuria	0.0001
LFT	0.415
Interaction proteinuria*LFT	0.637
Covariates	
Age	0.0001
Diabetes	0.874
Gender	0.376
Body Mass Index	0.619

Mixed Linear model with repeated measures of eGFR and covariates evaluated by AR (1) heterogeneous

Decline of eGFR was evaluated as repeated measure at time 1 (baseline) and time 2 (end of follow-up)

**Fig. 3:**
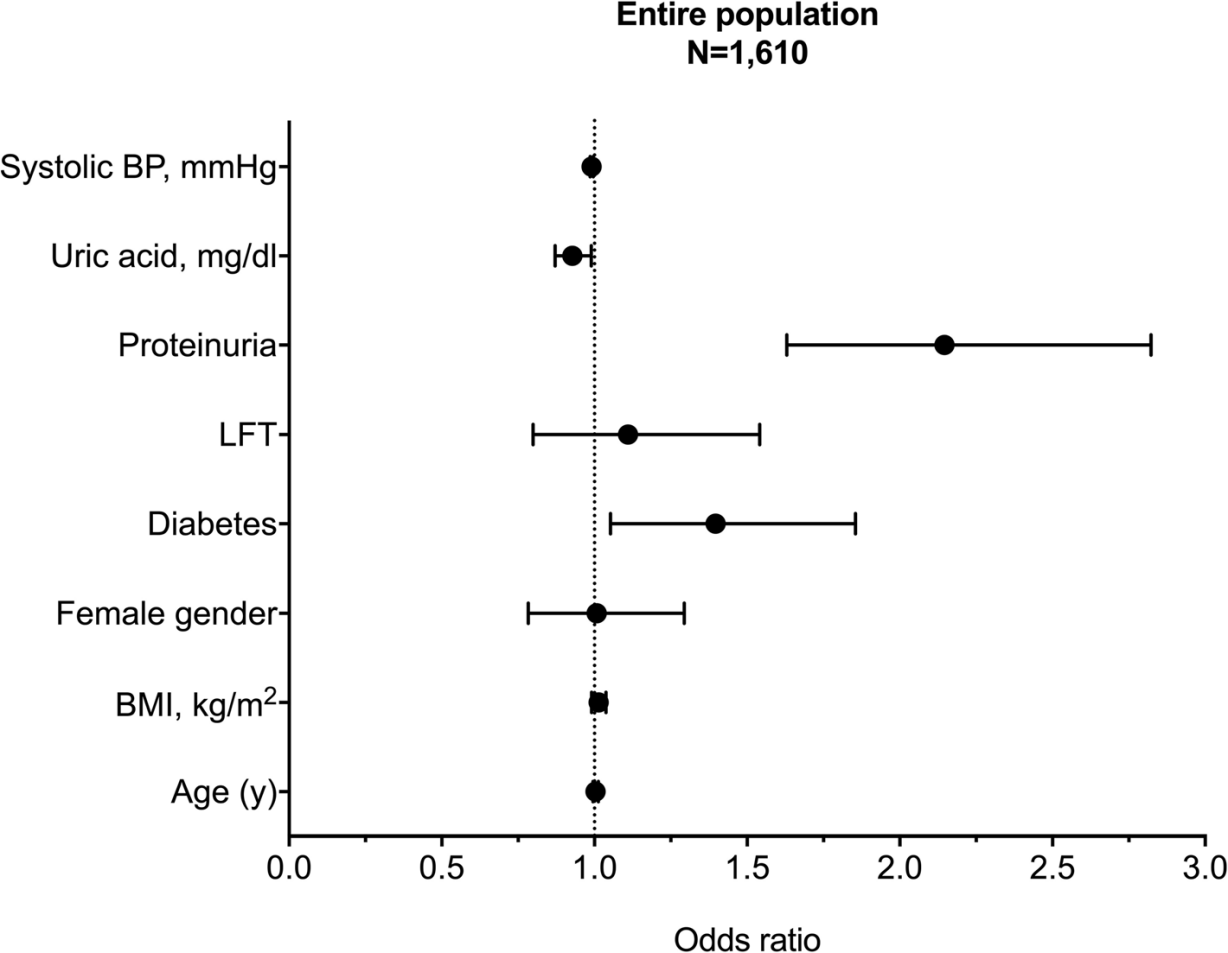
Odds ratio for decline of eGFR per month above − 0.05 ml/min/1.73m^2^. BP, blood pressure; LFT, low free thyroxin; BMI, body mass index

Logistic regression on the independent risk factors for eGFR/month decline was buil with the first quartile (worse eGFR decline) as the dependent variable. Results showed that proteinuria but not LFT was independently associated with a greater decline in renal function. Figure 3 shows the odds ratio (OR) and confidence interval (CI) of each independent variable in the logistic analysis.

CKD-EPI equation was calculated according to a previous report [18] and yielded similar results to MDRD (Table 1) in CKD progression.

## Discussion

In this retrospective analysis of CKD patients we failed to demonstrate an association between low thyroxin levels and rate of decline of kidney function. The sensitivity analysis, in patients with proteinuria was also negative, with no effect of LFT on eGFR decline, suggesting that LFT does not play a role on kidney function decline.

Based on experimental data, hypothyroidism could impact renal function decline as it promotes significant alterations on cardiac and renal systems, affecting glomerular filtration and renal plasma flow [21–24]. A few physiopathological processes can explain these finding. Firstly, a negative chronotropic and inotropic effect caused by bradycardia, with reduced myocardial contractility and reduced ventricular filling, can result in decreased cardiac output and as a consequence, activation of the renin-angiotensin-aldosterone system and decline in plasmatic levels of atrial natriuretic factor [25–27]. Secondly, pre-glomerular vasoconstriction can occur as an adaptive response to filtrate overload due to a deficiency of reabsorption of sodium and water by the proximal tubules [3]. Thirdly, a reduction in the expression of chloride channels on the basolateral membrane can affect chloride reabsorption and cause an increase of its concentration in the distal nephron activating tubule-glomerular feedback [3, 8]. Lastly, hypothyroidism has been associated with a reduction of insulin-like growth factor 1 (IGF1 and vascular endothelial growth factor (VEGF). IGF1 can promote increment of renal blood flow and creatinine clearance in humans, whereas VEGF augments endothelial nitric oxide synthase activity, enhancing the relaxing capability of renal vasculature [3, 28].

Despite this knowledge from experimental studies, clinical data demonstrating the association between LFT and loss of renal function is still controversial. Previous studies have shown that the decline in eGFR can be reversed, or at least attenuated, by exogenous thyroid hormone replacement, which indicates that kidney dysfunction could be the end result of functional changes rather than histological damage [7, 8]. While a recent large study showed, that there is an association between LFT and renal function deterioration and proteinuria [5], other large sample size studies either failed to demonstrated such association [11] or found opposite result [9]. These discrepancies could be explained by the design of the study (longitudinal vs. cross-sectional) and population enrolled (general vs. CKD). A community-based prospective cohort, the Atherosclerosis Risk in Communities study evaluated individuals with normal renal function and in a cross-sectional analysis did not find that thyroid dysfunction was related to CKD development [29]. A cross-sectional analysis of a healthy adult population in Brazil (mean eGFR 83.4 ml/min) showed that subclinical hypothyroidism (high levels of TSH) was associated with increased prevalence of CKD in euthyroid subjects [30]. In addition, the definition used for thyroid dysfunction is widely variable among studies, based on TSH, thyroxin levels, and/or symptoms. Kidney dysfunction is also highly variable among studies, include measurements of eGFR, serum creatinine, cystatin, or creatinine clearance, making the comparison of the results challenging.

It is well accepted that proteinuria is associated with worse renal and clinical outcomes, including myocardial infarction, and all-cause mortality [21]. Proteinuria and albuminuria are also markers of kidney damage, and associated with eGFR decline and progression to ESRD [31, 32]. Our data confirmed proteinuria as a marker of impairment of renal function in the long term follow up.

There are some limitations to our study. First, it is a retrospective study, and we did not control for the frequency of thyroid function and creatinne assessments, as they were measured at the discretion of health care providers. Secondly, we used creatinine-based estimates of eGFR and not assessed measured GFR; in aditiion we used Jaffé assay to measure creatinine, which is less accurate in the presence of glucose or protein. Patient adherence to thyroid hormone supplementation and dose were not available. We have focused on the final eGFR to calculate decline, and our follow-up was relatively short. Lastly, we did not have information on the coexisting medical conditions such as cardiac disease or neoplasia [33]. Nevertheless, we believe we have a large enough sample size that reduces the effect of these flaws.

## Conclusions

Our study failed to demonstrated an association between low free thyroxine levels and CKD progression. The rate of progression of the CKD was greater in the presence of proteinuria, with no effect of thyroxine levels. Given the emerging literature on the association between LFT and loss of kidney function, our findings indicate the need for a prospective studies evaluating the impact of hypothyroidism treatment on CKD progression.

## Data Availability

The dataset analysed during the current study are available from the corresponding author on reasonable request.

## Abbreviations

AKI: Acute kidney injury
BMI: Body mass index
CKD: Chronic kidney disease
eGFR: Estimated glomerular filtration rate
fT4: Free thyroxine
IGF1: Insulin-like growth factor 1
LFT: Low free thyroxine
MDRD: Modification of Diet in Renal Disease
PCR: Protein-creatinine ratio
sCr: Serum creatinine
TSH: Thyroid stimulating hormone
VEGF: Vascular endothelial growth factor
